# Nano‑zinc enhances gene regulation of non‑specific immunity and antioxidative status to mitigate multiple stresses in fish

**DOI:** 10.1038/s41598-023-32296-y

**Published:** 2023-03-28

**Authors:** Neeraj Kumar, Dilip Kumar Singh, Nitish Kumar Chandan, Supriya Tukaram Thorat, Pooja Bapurao Patole, Archana Gite, Kotha Sammi Reddy

**Affiliations:** 1 ICAR-National Institute of Abiotic Stress Management, Malegaon, Baramati, Pune, 413115 India; 2 ICAR-Central Institute of Fisheries Education, Kolkata Center, Kolkata, 700091 India; 3 ICAR-Central Institute of Freshwater Aquaculture, Bhubaneshwar, 751002 India

**Keywords:** Ichthyology, Metabolism

## Abstract

The toxicity of ammonia surged with arsenic pollution and high temperature (34 °C). As climate change enhances the pollution in water bodies, however, the aquatic animals are drastically affected and extinct from nature. The present investigation aims to mitigate arsenic and ammonia toxicity and high-temperature stress (As + NH_3_ + T) using zinc nanoparticles (Zn-NPs) in *Pangasianodon hypophthalmus.* Zn-NPs were synthesized using fisheries waste to developing Zn-NPs diets. The four isonitrogenous and isocaloric diets were formulated and prepared. The diets containing Zn-NPs at 0 (control), 2, 4 and 6 mg kg^−1^ diets were included. Superoxide dismutase (SOD), catalase (CAT), glutathione peroxidase (GPx) and glutathione-s-transferase (GST) were noticeably improved using Zn-NPs diets in fish reared under with or without stressors. Interestingly, lipid peroxidation was significantly reduced, whereas vitamin C and acetylcholine esterase were enhanced with supplementation of Zn-NPs diets. Immune-related attributes such as total protein, globulin, albumin, myeloperoxidase (MPO), A:G ratio, and NBT were also improved with Zn-NPs at 4 mg kg^−1^ diet. The immune-related genes such as immunoglobulin (Ig), tumor necrosis factor (TNFα), and interleukin (IL1b) were strengthening in the fish using Zn-NPs diets. Indeed, the gene regulations of growth hormone (GH), growth hormone regulator (GHR1), myostatin (MYST) and somatostatin (SMT) were significantly improved with Zn-NPs diets. Blood glucose, cortisol and HSP 70 gene expressions were significantly upregulated by stressors, whereas the dietary Zn-NPs downregulated the gene expression. Blood profiling (RBC, WBC and Hb) was reduced considerably with stressors (As + NH_3_ + T), whereas Zn-NPs enhanced the RBC, WBC, and Hb count in fish reread in control or stress conditions. DNA damage-inducible protein gene and DNA damage were significantly reduced using Zn-NPs at 4 mg kg^−1^ diet. Moreover, the Zn-NPs also enhanced the arsenic detoxification in different fish tissues. The present investigation revealed that Zn-NPs diets mitigate ammonia and arsenic toxicity, and high-temperature stress in *P. hypophthalmus*.

## Introduction

Climate change and pollution are the major drivers for degrading the aquatic ecosystem. The aquatic systems receive inorganic and organic contaminants from different sources affecting the aquatic organisms. It is a great question mark that how aquatic organisms will be exist in the future as the impact of climate change and pollution is increasing day to day. IPCC revealed that global warming is likely to increase by 1.5 °C during 2030–2052, if it continues to increase at the current rate^1^. Selection of suitable species, dietary manipulation, and local adaptation may answer the following question. However, arsenic contamination in an aquatic system, excessive ammonia production in culture systems, and high temperature stress due to global climate change are the natural problems in the culture systems. Which may be affected by species extinction and hamper the productivity of culture and capture systems. Interestingly, total ammonia is present in two forms, non-ionized (NH_3_) and ionized (NH_4_^+^), and NH_3_ induces and or diffuses the toxicity through biological cell membrane^2,3^. The fish excreta and un-eaten feed is the major source of ammonia in aquaculture^4–6^. It is also most toxic for fish. Its impairment in vital organs causes mass mortality of aquatic animals by its toxicity^7^. However, its toxicity also reduced the growth performance, physiology alteration, feeding regime, immune system and enhanced the oxidative stress and apoptosis in fish^5,7–9^.

The water bodies are also contaminated with arsenic pollution worldwide which affects aquatic animals. Arsenic has also dominated foodstuffs, ground, and drinking water, a major public concern globally^10,11^. More than 100 countries were reported arsenic groundwater contamination including India, USA, China, Bangladesh, Indonesia, and other parts of the word^12–15^. Further, the arsenic contamination was enhanced with the combination of other stressors such as ammonia and high-temperature stress in aquatic animals, including fish. The other contaminants greatly surge the toxicity of arsenic and affected the fish due to enhancing the stresses in fish. It is very difficult for the fish to adapt or mitigate the stressors in combination with arsenic, ammonia toxicity, and high-temperature stress.

The aquaculture industries need technologies to revamp in the form of nano-based products such as the Zn-NPs diet, which reduces the cost of the feed and cleans the aquatic environments^16^. Zinc is the best nutrient for nano-based feed formulation as it stands in the third position among the different nanoparticles produced annually^17^. It has better antibacterial properties^18^, as well as a better anabolic agent, immune enhancer, reproduction, cell proliferation, defense against free radicals, and biochemical reactions as a co-factor or precursor^19–23^. It has also an important role as a cofactor for metalloenzymes such as carboxypeptidase, alcohol dehydrogenase, carbonic anhydrase, glutamic dehydrogenase, and D-superoxide dismutase^24^. It is also noted that Zn-NPs and Zn compounds have a similar zinc to oxygen ratio, whereas, at the nano level, atoms are arranged with a wider energy level confinement with small space^19^.

The present investigation was conducted in *Pangasianodon hypophthalmus*, a stress tolerant, and fast-growing fish^20–22^. It also has high market demand and suitable candidate species for the diversification of aquaculture^23^. Moreover, the stressors such as arsenic, ammonia, and high temperature (As + NH_3_ + T) disrupt the biological macromolecules, cell signaling, and genes involved in protecting fish against stressors. It also reduces the anti-oxidative status (superoxide dismutase, catalase, glutathione peroxidase, and glutathione-s-transferase) and enhances the lipid peroxidation in the cell^25^. Arsenic, ammonia, and high temperature also disrupt the several genes involved in immunity, defense mechanism, oxidative stress, and growth performance. Therefore, the present investigation aimed to evaluate the protective role of Zn-NPs against multiple stresses (arsenic pollution, ammonia toxicity, and high temperature stress) in fish.

## Material and methods

### Ethics statement

In the present study, we strictly followed the guideline of ARRIVE (Animal Research: Reporting of In Vivo Experiments). The methodology and end-points of the results were approved by the Director, NIASM and RAC (Research Advisory Committee). We also followed the International and National Guidelines for caring the animal during the experiment.

### Experimental animal and design

*P. hypophthalmus* weighing 5.74 ± 0.36 g and length of 5.12 cm was used in this study. The fish were reared in the NIASM farm pond for experimental purposes. The fish were fed with practical diet containing 30% protein during the rearing period for 2 months before commencing the experiment. Eight treatments (8) with three replicates and each replicates containing eighteen fish (18) were used for this experiment. The details of experimental designs are presented in Table 1.

**Table 1:** Details of experimental designed.

Treatment number (s)	Symbols	Treatments	Treatments details
1	Control	Control group	No exposure to stressors and fed with control diet
2	As + NH_3_ + T	Arsenic (2.68 mg L^−1^), ammonia (2.0 mg L^−1^) and high temperature (34 °C) exposure	Concurrent exposure to arsenic (2.68 mg L^−1^), NH_3_ (2.0 mg L^−1^) and high temperature (34 °C) and fed with control diet
3	Zn-NPs-2.0 mg kg^−1^	Zn-NPs at 2.0 mg kg^−1^ diet	Fed with Zn-NPs at 2.0 mg kg^−1^
4	Zn-NPs-4.0 mg kg^−1^	Zn-NPs at 4.0 mg kg^−1^ diet	Fed with Zn-NPs at 4.0 mg kg^−1^
5	Zn-NPs-6.0 mg kg^−1^	Zn-NPs at 6.0 mg kg^−1^ diet	Fed with Zn-NPs at 6.0 mg kg^−1^
6	Zn-NPs-2.0 mg kg^−1^As + NH_3_ + T	Zn-NPs at 2.0 mg kg^−1^ diet along with stressors group	Fed with Zn-NPs at 2.0 mg kg^−1^ and concurrent exposure to arsenic (2.68 mg L^−1^), NH_3_ (2.0 mg L^−1^) and high temperature (34 °C)
7	Zn-NPs-4.0 mg kg^−1^As + NH_3_ + T	Zn-NPs at 4.0 mg kg^−1^ diet along with stressors group	Fed with Zn-NPs at 4.0 mg kg^−1^ and concurrent exposure to arsenic (2.68 mg L^−1^), NH_3_ (2.0 mg L^−1^) and high temperature (34 °C)
8	Zn-NPs-6.0 mg kg^−1^As + NH_3_ + T	Zn-NPs at 6.0 mg kg^−1^ diet along with stressors group	Fed with Zn-NPs at 6.0 mg kg^−1^ and concurrent exposure to arsenic (2.68 mg L^−1^), NH_3_ (2.0 mg L^−1^) and high temperature (34 °C)

The aeration was provided throughout the experiment using an aerator pump. The water quality was analyzed periodically as per American Public Health Association (APHA)^26^. Two third of (2/3rd) experimental water was manually exchanged on every alternate day. The faecal matters and uneaten diets were removed using siphoning in each tank daily. The arsenic (1/10th of LC_50_ 2.68 mg L^−1^ of arsenic)^10^, ammonia sulphate as NH_3_ (1/10th of LC_50_ 2.0 mg L^−1^ of NH_3_) (TAN)^27^ and high temperature (34 °C) were maintained in the stressors group.

### Experimental diets

The iso-nitrogenous (35% crude protein) and iso-caloric (394 kcal/100 g) diets were prepared and graded with Zn-NPs at 0, 2, 4, and 6 mg kg^−1^ diets. During feed preparation the different ingredients were used such as fish meal, groundnut meal, soybean meal, wheat flour, carboxymethyl cellulose (CMC), cod liver oil, lecithin, and vitamin C. Zinc free vitamin mineral mixture was prepared manually for inclusion in the diet. The heat labile ingredients were mixed after heating the feed ingredient. Zn-NPs were not incorporated in the control diet, whereas the other three diets were mixed Zn-NPs. Proximate analysis of the diets was analyzed using the AOAC method^28^. Crude protein was analyzed using nitrogen content, ether extract (EE) using solvent extraction and Ash estimation using a muffle furnace (550 °C) (Table 2). Total carbohydrate % was calculated using the following equation:

$${\text{Total}}\;{\text{carbohydrate}}\% = 100 - \left( {{\text{Moisture}} + {\text{CP}}\% + {\text{EE}}\% + {\text{Ash}}\% } \right).$$

**Table 2:** Ingredient composition and proximate analysis of experimental diets (% dry matter) of zinc nanoparticles (Zn-NPs), fed to *Pangasianodon hypophthalmus* during the experimental period of 105 days.

Feed ingredients	Zn-NPs-0 mg kg^−1^ diet	Zn-NPs-2 mg kg^−1^ diet	Zn-NPs-4 mg kg^−1^ diet	Zn-NPs-6 mg kg^−1^ diet
Soybean meal^a^	35.5	35.5	35.5	35.5
Fish meal^a^	25	25	25	25
Groundnut meal^a^	10	10	10	10
Wheat flour^a^	17.2	17.198	17.196	17.194
Sunflower oil^a^	4.5	4.5	4.5	4.5
Cod liver oil^a^	1.5	1.5	1.5	1.5
CMC^b^	2	2	2	2
Vitamin and mineral mix*^c^	2	2	2	2
Vitamin C^d^	0.3	0.3	0.3	0.3
Lecithin^b^	2	2	2	2
Zn-NPs	0	0.002	0.004	0.006
Proximate composition of the diets				
CP^1^	35.31 ± 0.07	35.60 ± 0.36	35.43 ± 0.42	35.28 ± 0.09
EE^2^	9.15 ± 0.05	9.24 ± 0.03	9.43 ± 0.04	9.28 ± 0.02
TC^3^	47.36 ± 0.17	46.67 ± 0.50	46.81 ± 0.43	47.13 ± 0.01
OM^4^	91.82 ± 0.10	91.51 ± 0.13	91.66 ± 0.07	91.69 ± 0.06
DM^5^	91.90 ± 0.13	92.36 ± 0.30	92.74 ± 0.04	91.83 ± 0.13
DE^6^	394.71 ± 0.42	393.75 ± 0.47	394.92 ± 0.38	394.61 ± 0.16

^a^Procured from local market, ^b^Himedia Ltd, ^c^*Prepared manually and all components from Himedia Ltd, ^d^SD Fine Chemicals Ltd., India.

*Manual prepared Vitamin mineral mixture; Composition of vitamin mineral mix (quantity/250 g starch powder): vitamin A 55,00,00 IU; vitamin D3 11,00,00 IU; vitamin B1:20 mg; vitamin E 75 mg; vitamin K 1,00 mg; vitamin B12 0.6 mcg; calcium pantothenate 2,50 mg; nicotinamide 1000 mg; pyridoxine: 100 mg; Mn 2700 mg; I 1,00 mg; Fe 750 mg; Cu 200 mg; Co 45 mg; Ca 50 g; P 30 g; Se: 2 ppm.

*CP*^1^ Crude Protein, *EE*^2^ Ether extract, *TC*^3^ Total Carbohydrate, *OM*^4^ Organic Matter, *DM*^5^ Dry matter, *DE*^6^ Digestible Energy.

Digestible energy (DE) (Kcal/100 g) = (% CP × 4) + (% EE × 9) + (TC × 4).

Data expressed as mean ± SE, *n* = 3.

The gross energy of the diets was calculated using method described by Halver method^29^.

### Green synthesis and characterization of Zn-NPs

**Figure 1:**
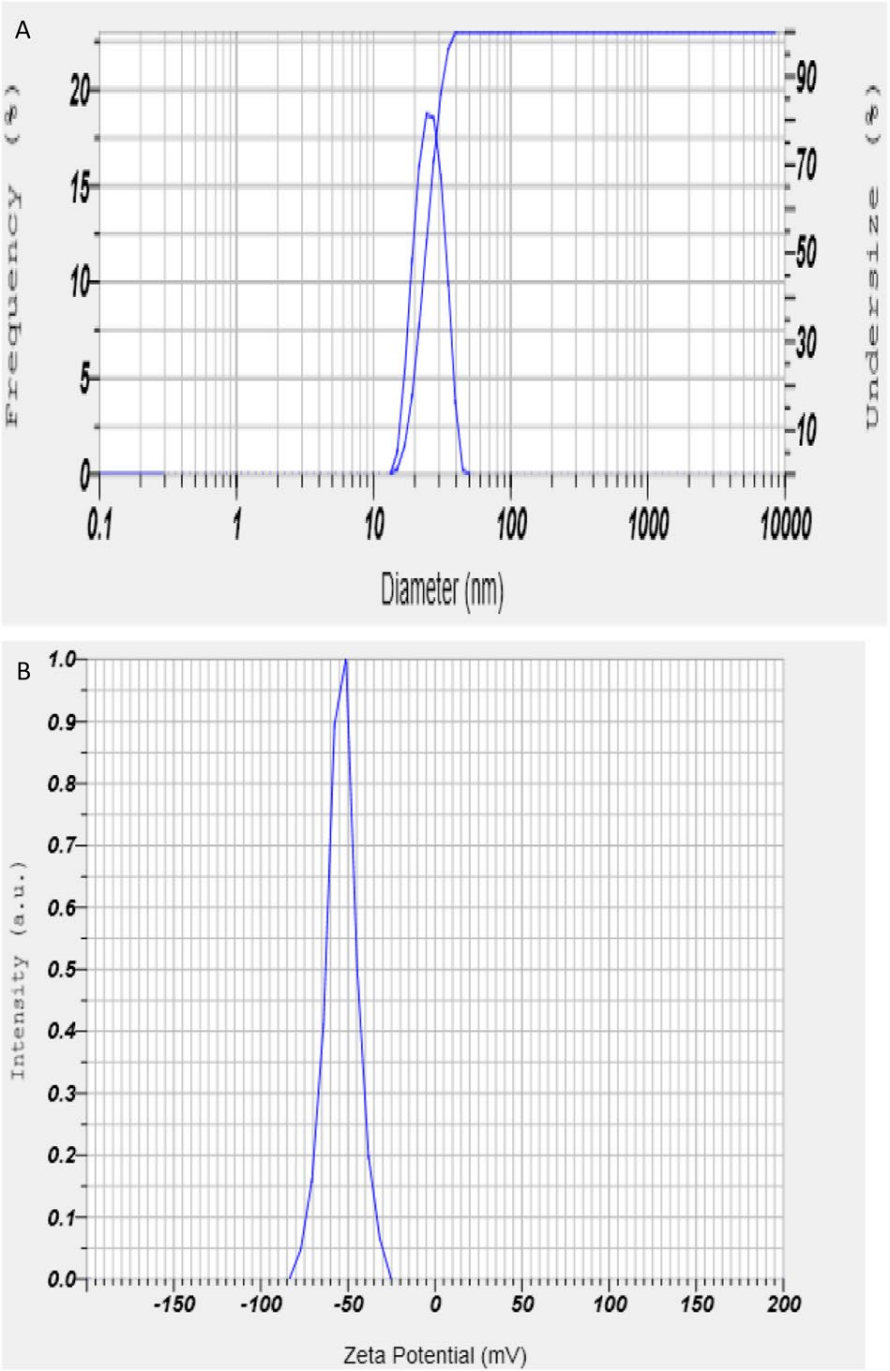
(**A**,**B)** Size (23 nm) and zeta potential (− 53.7 mV) of zinc-nanoparticles.

Zn-NPs was synthesized using our previous method^20,21,30^. The primary characterization of Zn-NPs was performed using a spectrophotometer at 300–500 nm. The peak during characterization was obtained in the 260–380 nm range. The zeta potential and mean size were obtained at 53.7 mV and 23 nm, respectively (Fig. 1A,B) (Particle Analyser, Litesizer 500, Anton Paar, Austria).

### Sample preparation for enzymatic analysis and blood collection

Four fish were used for the collection of tissues, such as the liver, gill, kidney, and brain, an aseptic condition after 105 days of experimental periods. The fish were anesthetizing using clove oil at 100 µg L^−1^ during dissection. The tissues were homogenized using a homogenizer (Omni Tissue Master Homogenize, Kennesaw, GA) with EDTA (1 mM) in 0.25 M chilled sucrose solution. Then homogenized tissues were centrifuged at 6000 xg for 20 min at 4 °C. The supernatants were kept at − 80 °C for further analysis. The blood was drawn from all four fish, and additionally, four fish were used for the collection of blood and serum for analysis of immune-related attributes before collecting the tissues. Blood was drawn from the caudal peduncle region (*v. caudalis*) using heparinized syringe (Heparin Injection, 5000 IU heparin sodium salt in 1 ml). Erythrocyte count (RBC), hemoglobin (Hb), WBC (total leucocyte count) were determined using Svobodova method^31^. The tissue protein was determined by the Lowry method^32^.

### RNA isolation and quantification

Total RNA was isolated from muscle tissue of *P. hypophthalmus* using TRIZOL reagent (Ambion, as per manufacturer's directions). Muscle tissues of 50 mg homogenized in liquid nitrogen using mortar and pestle and lysed in TRIzol reagent. It was incubated for 5 min after adding chloroform for phase separation. After centrifugation, the aqueous phase containing RNA separated in 1.5 ml tube, then RNA precipitated using isopropanol. The precipitated RNA was washed with 75% ethanol, and air-dried RNA pellet was dissolved in RNAse free water. Moreover, RNA was stored at – 80 °C for further use. The RNA bands were visualized in a gel documentation system (ChemiDoc^TM^ MP imaging system, Bio-Rad). The RNA was quantified using Nano-Drop spectrophotometer (Thermo-scientific). 

### cDNA synthesis and quantitative PCR

Total isolated RNA was used for cDNA synthesis using Revert Aid First strand cDNA synthesis kit (Thermo-scientific). DNase I was used to removing trace amounts of DNA before cDNA synthesis. RNA (100 ng) and oligo dT primers (15 pmol) was placed in 12 µl volume. The reaction mixture was heated at 65 °C for 5 min in PCR and then chilled on ice. After that, 1 µl Ribo Lock RNase Inhibitor (20 U/µL), 1.0 µl of reverse transcriptase enzyme, 5 X reaction buffer (4.0 µl) and 2 µl dNTP Mix (10 mM) were added to the chilled mixture, followed by a centrifuge for few second. After that, the mixture was incubated at 60 °C for 42 min and then at 70 °C for 5 min, and stored the synthesized cDNA at − 20 °C. The synthesized cDNA was confirmed using β-actin PCR. Gene-specific primers were used to perform quantitative PCR (Real time PCR) using SYBR green PCA master mix (Bio-Rad). SYBR Green Master Mix (1X), primer (1 µl), 1 µl of c-DNA were mixed and setup for the reaction cycle as initial denaturation at 95 °C for 10 min, amplification of the c-DNA for 39 cycle and then denaturation for 15 s at 95 °C and annealing for 1 min at 60 °C^33^ for quantifications.

### Growth, anti-oxidative and immune-related gene study

The growth, immunity, and anti-oxidative related genes were investigated in this study. Heat shock protein (HSP 70), nitric oxide synthase (iNOS), tumor necrosis factor (TNFα), growth hormone receptor (Ghr1 and Ghrb), interleukin (IL1β), immunoglobulin (Ig), growth hormone (GH), somatostatin (SMT) and myostatin (MYST) were studied for real time quantification. The details of the primers are shown in Table 3.

**Table 3:** Details of primer for relative quantitative real-time PCR.

Gene	Primer sequence (5′–3′)	Accession number
HSP 70	F-CTCCTCCTAAACCCCGAGTC R-CCACCAGCACGTTAAACACA	XM_026934573.2
iNOS	F-ACACCACGGAGTGTGTTCGT R-GGATGCATGGGACGTTGCTG	XM_026931613.2
DNA Damage	F-CACCTTCGCCCTCGAAGTCT R-GCTCGGGTGAGGTCTCTCAG	XM_026938137.2
TNFα	F-TGGAGTTCTGCTTGCCGTGG R-GCAGCCTTTGCAGTCTCGGA	XM_026942329.2
Ghr1	FTATTGGCTACAGCTCGCCGC R-AATCACCCCGACTGTGCTGC	XM_034306157.1
Ghrb	F-TTGAGCTTTGGGACTCGGAC R-CGTCGATCTTCTCGGTGAGG	XM_026942987.2
IL1b	F- AGCAGGATCCATCAAAGTGG R- GTGCTCCAGCTCTCTGGGTA	XM_026918084.2
Ig	F- GGCCAGTAATCGTACCTCCA R- CTTCGTAAGGTCCCCACTGA	XM_026923540.2
Myostatin	F-GGGAAAGACCTGGCCGTGAC R-TCGAGGCCGGATTCTCGTCT	XM_026910492.2
SMT	F-CTCTGGGTGGCAGAATGAAT R-AACATGAAGAGAACGTTTTCCAG	XM_026921272.2
GH	F-CCCAGCAAGAACCTCGGCAA R-GCGGAGCCAGAGAGTCGTTC	GQ859589.1
β-Actin	F-CAGCAAGCAGGAGTACGATG R-TGTGTGGTGTGTGGTTGTTTTG	XM_031749543.1

*HSP* Heat shock protein, *iNOS* Nitric oxide synthase, *TNF* Tumor necrosis factor, *Ghr* Growth hormone receptor, *IL* Interleukin, *Ig* Immunoglobulin, *SMT* Somatostatin, *GH* Growth hormone.

### Oxidative stress enzymes

Catalase (EC 1.15.1.1) activity was determined as per Takahara et al.^34^. Glutathione-S-transferase (GST) (EC 2.5.1.18) activities were determined at 412 nm as per Habing et al.^35^, Paglia and Valentine^36^ was applied to measure the glutathione peroxidase (GPx) activities. Similarly, Misra and Fridovich^37^ were used to determine the superoxide dismutase (SOD) (EC 1.15.1.1) activity.

### Neurotransmitter enzyme activities

The acetylcholine esterase (AChE, EC. 3.1.1.7)) activities in brain tissue was determined using Hestrin modified by the Augustinsson^38^ method.

### Lipid peroxidation (LPO) and Vitamin C

Uchiyama and Mihara^39^ [41] method was followed to determine the LPO in liver and kidney tissues. Similarly, Roe and Keuther^40^ applied this to determine the Vitamin C in brain and muscle tissues.

### Immunological attributes

Total serum protein, albumin, globulin, and A:G ratio was determined using the protein estimation kit. Secombes^41^, with some modification by Stasiack and Baumann^42^ used to estimate respiratory burst activity. The blood glucose was determined using Nelson^43^ and Somoyogi^44^. Moreover, Quade and Roth^45^, with some modifications^46^ and Anderson and Siwicki^47^ were applied to determine myeloperoxidase and total immunoglobulin.

### Cortisol and HSP-70

HSP 70 and cortisol were performed using ELISA kit (Bioguenix/Enzo Life Science, Mumbai, India) and (Cayman Chemicals, USA).

### Alkaline single-cell gel electrophoresis (SCGE)/Comet assay

DNA damage was carried out by using alkaline single-cell gel electrophoresis. The comet assay was performed in kidney tissue by three agarose layers with slight modification^48^. The slides were analyzed in a fluorescent microscope (Leica Microsystems Ltd, DM 2000, Heerbrugg, Switzerland) and then captured photographs and analyzed in an image analysis system with Open comet. DNA damage was quantified as percent tail DNA (% tail DNA = 100% head DNA).

### Growth performance

The growth performance sampling was performed on every 15 days intervals until the end of the 105 days experiment. Final body weight gain (FWG %), protein efficiency ratio (PER), specific growth rate (SGR), feed efficiency ratio (FER), daily growth index (DGI %), thermal growth coefficient, and relative feed intake were followed to determine the growth performance of the fish.

$${\text{Final}}\;{\text{weight}}\;{\text{gain}}\left( \% \right) = {\text{Final}}\;{\text{body}}\;{\text{weight}}\left( {{\text{FBW}}} \right) - {\text{Initial}}\;{\text{body}}\;{\text{weight}}\left( {{\text{IBW}}} \right)/{\text{Initial}}\;{\text{body}}\;{\text{weight}}\left( {{\text{IBW}}} \right) \, \times 100$$

$${\text{SGR}} = 100\left( {\ln \;{\text{FBW}} - \ln \;{\text{IBW}}} \right)/{\text{number}}\;{\text{of}}\;{\text{days}}$$

$${\text{PER}} = {\text{Total}}\;{\text{wet}}\;{\text{weight}}\;{\text{gain}}\left( {\text{g}} \right)/{\text{crude}}\;{\text{protein}}\;{\text{intake}}\left( {\text{g}} \right)$$

$${\text{FER}} = {\text{Wet}}\;{\text{weight}}\;{\text{gain}}\left( {\text{g}} \right)/{\text{total}}\;{\text{dry}}\;{\text{feed}}\;{\text{intake}}\left( {\text{g}} \right)$$

$${\text{Daily}}\;{\text{growth}}\;{\text{index}},{\text{DGI}}\left( \% \right) = \left( {{\text{FBW}}1/3{-}{\text{IBW}}1/3} \right)/{\text{days }} \times \, 100$$

$${\text{Relative}}\;{\text{feed}}\;{\text{intake}},\left( {{\text{FI}}} \right)\left( {\% /d} \right) = 100 \times \left( {{\text{TFI}}/{\text{{\rm I}BW}}} \right)$$

$${\text{Thermal}}\;{\text{growth}}\;{\text{coefficient}},\left( {{\text{TGC}}} \right) = \left( {{\text{FBW}}1/3{-}{\text{IBW}}1/3} \right) \times \left( {\Sigma {\text{D}}0} \right) - 1,$$

where ΣD0 is the thermal sum (feeding days × average temperature, °C).

### Sample preparation for arsenic analysis

The bioaccumulation of arsenic in different fish tissues and experimental water was analyzed using our previous report with Inductively Coupled Plasma Mass Spectrometry (ICPMS, Agilent 7700 series, Agilent Technologies, USA)^49,50^.

### Statistical analysis

The data were analyzed using one-way analysis of variance (ANOVA) with Duncan’s multiple range tests (DMRT) (SPSS 16, Chicago, IL, USA). Before ANOVA, the data were analyzed for homogeneity and normality of variance using Levene’s and Shapiro–Wilk’s tests, respectively. The data were noted as mean ± standard error.

## Results

### Characterisation of Zn-NPs

The Zn-NPs were synthesized using biological approaches as fisheries waste (fish gill). The primary characterization of Zn-NPs was performed using UV spectrophotometer in the range of 200–500 nm, which the peak obtained at 260–380 nm. The size and zeta potential were determined using a Nanoparticle size analyser. The size and zeta potential were obtained as 23 nm and − 53.7 mV, respectively (Fig. 1A,B).

### Zn-NPs on anti-oxidative status

The anti-oxidative status viz. SOD, GST, GPx, and CAT in gill, liver and kidney tissues against ammonia and arsenic toxicity and high temperature stress in *P. hypophthalmus* and data are recorded in the Tables 4 and 5. CAT activities in the liver (*p* = 0.0023), gill (*p* = 0.0016), and kidney (*p* = 0.0011) tissues were noticeably reduces on supplementation of Zn-NPs at 4 mg kg^−1^ diet followed by Zn-NPs at 2 mg kg^−1^ diet in comparison to control and stressor group. Whereas, CAT activities were significantly higher with exposure to As + NH_3_ + T compared to control and Zn-NPs diet with or without stressors (Table 4). Further, the SOD activities in the liver (*p* = 0.032) and kidney (*p* = 0.042) were remarkably higher in the group treated with As + T + NH_3,_ in comparison to control and Zn-NPs at 4 mg kg^−1^. SOD activities were significantly (*p* < 0.01) reduced with supplementation of Zn-NPs at 4 mg kg^−1^ diet compared to control and stressor groups. However, SOD activities in kidney tissue were non-significant among all the treatments (*p* > 0.05) (Table 4). GST activities in the liver (*p* = 0.0062), gill (*p* = 0.018), and kidney (*p* = 0.024) tissues were noticeably decreases with supplementation of Zn-NPs at 4 mg kg^−1^ diet. Whereas GST activities were significantly elevated with As + NH_3_ + T compared to control and Zn-NPs supplemented group (Zn-NPs at 4 mg kg^−1^ diet) (Table 4). The activities of GPx in the liver (*p* = 0.0013), gill (0.0032) and kidney (*p* = 0.0036) tissues were significantly elevated with As + T + NH_3_, in comparison to all the experimental groups. Whereas dietary Zn-NPs at 4 mg kg^−1^ diet noticeably reduced the GPx activities compared to control and other groups with or without stressors (Table 5). Zn-NPs at 6 mg kg^−1^ diet was not improve the anti-oxidative status of the fish reared under stress or without stress.

**Table 4:** Effect of dietary Zn-NPs on catalase, superoxide dismutase and glutathione-s-transferase in liver, gill and kidney tissues of *P. hypophthalmus* reared under control condition or arsenic, ammonia and high temperature stress for 105 days.

Treatments	Non-Stressors	Stressors (As + NH_3_ + T)	Non-Stressors			Stressors (As + NH_3_ + T)			*P*-Value
Diets	Control	Control	Zn-NPs-2	Zn-NPs-4	Zn-NPs-6	Zn-NPs-2	Zn-NPs-4	Zn-NPs-6	
CAT-L	7.11 ± 0.38^b^	21.30 ± 0.87^d^	6.55 ± 0.58^b^	4.22 ± 0.71^a^	8.17 ± 1.09^c^	7.09 ± 0.99^b^	4.73 ± 0.60^a^	10.22 ± 1.0^d^	0.0023
CAT-G	6.39 ± 0.51^b^	19.46 ± 1.25^e^	6.24 ± 0.74^b^	3.77 ± 0.24^a^	8.75 ± 0.89^c^	6.35 ± 0.67^b^	4.43 ± 0.56^a^	11.38 ± 1.49^d^	0.0016
CAT-K	6.86 ± 1.17^b^	17.69 ± 1.61^e^	6.63 ± 0.64^b^	3.34 ± 0.82^a^	9.22 ± 0.74^c^	7.13 ± 0.49^b^	2.76 ± 0.53^a^	12.16 ± 1.33^d^	0.0011
SOD-L	56.22 ± 1.35^b^	64.54 ± 0.46^e^	58.25 ± 1.24^c^	52.53 ± 0.56^a^	57.24 ± 1.40^bc^	59.49 ± 0.8^c^	54.73 ± 0.61^ab^	61.60 ± 0.71^d^	0.032
SOD-G	41.57 ± 1.70	45.53 ± 2.62	42.67 ± 1.35	40.01 ± 0.77	42.99 ± 2.62	43.97 ± 2.10	39.71 ± 0.39	47.59 ± 0.74	0.082
SOD-K	64.17 ± 3.87^b^	65.26 ± 0.37^c^	66.05 ± 1.43^c^	58.79 ± 0.99^a^	69.55 ± 1.35^d^	69.59 ± 1.92^d^	59.58 ± 0.24^a^	69.29 ± 1.35^d^	0.042
GST-L	0.22 ± 0.03^b^	0.77 ± 0.04^d^	0.22 ± 0.03^b^	0.14 ± 0.01^a^	0.35 ± 0.02^c^	0.29 ± 0.03^bc^	0.13 ± 0.01^a^	0.39 ± 0.05^c^	0.0062
GST-G	0.29 ± 0.03^b^	0.69 ± 0.02^d^	0.35 ± 0.01^c^	0.14 ± 0.01^a^	0.31 ± 0.02^b^	0.34 ± 0.03^c^	0.17 ± 0.02^a^	0.38 ± 0.03^c^	0.018
GST-K	0.24 ± 0.03^b^	0.54 ± 0.02^d^	0.28 ± 0.01^b^	0.15 ± 0.01^a^	0.38 ± 0.02^c^	0.25 ± 0.03^b^	0.15 ± 0.01^a^	0.39 ± 0.04^c^	0.024

Values in the same columns with different superscript (a, b, c, d, e) differ significantly. Data expressed as Mean ± SE (n = 6). CAT, SOD, and GST Units/mg protein.

**Table 5:** Effect of dietary Zn-NPs on glutathione peroxidase, lipid peroxidation, acetylcholine esterase and vitamin C in *P. hypophthalmus* reared under control condition or arsenic, ammonia and high temperature stress for 105 days.

Treatments	Non-Stressors	Stressors (As + NH_3_ + T)	Non-Stressors			Stressors (As + NH_3_ + T)			*P*-Value
Diets	Control	Control	Zn-NPs-2	Zn-NPs-4	Zn-NPs-6	Zn-NPs-2	Zn-NPs-4	Zn-NPs-6	
GPx-L	0.45 ± 0.02^b^	0.80 ± 0.07^d^	0.42 ± 0.05^b^	0.26 ± 0.01^a^	0.64 ± 0.03^c^	0.44 ± 0.03	0.30 ± 0.02^a^	0.60 ± 0.03^c^	0.0013
GPx-G	0.37 ± 0.02^b^	0.72 ± 0.06^d^	0.37 ± 0.03^b^	0.25 ± 0.02^a^	0.47 ± 0.04^c^	0.43 ± 0.02^d^	0.21 ± 0.01^a^	0.48 ± 0.06^c^	0.0032
GPx-K	0.40 ± 0.04^b^	0.79 ± 0.05^d^	0.62 ± 0.03^c^	0.30 ± 0.02^a^	0.57 ± 0.05^c^	0.65 ± 0.03^c^	0.33 ± 0.03^a^	0.62 ± 0.03^c^	0.0036
LPO-L	17.52 ± 0.44^b^	34.22 ± 0.55^d^	17.16 ± 0.43^b^	13.58 ± 0.71^a^	22.97 ± 0.54^c^	21.27 ± 0.67^c^	14.99 ± 0.66^a^	23.27 ± 0.23^c^	0.0011
LPO-K	21.65 ± 0.54^b^	41.77 ± 0.86^d^	21.82 ± 1.48^b^	13.65 ± 0.52^a^	25.70 ± 0.59^c^	21.14 ± 0.97^b^	14.11 ± 1.02^a^	21.64 ± 0.62^b^	0.0018
AChE	0.47 ± 0.03^cd^	0.26 ± 0.01^a^	0.50 ± 0.04^d^	0.63 ± 0.05^e^	0.42 ± 0.02^c^	0.47 ± 0.05^cd^	0.69 ± 0.06^f^	0.39 ± 0.02^b^	0.0046
VitC-M	19.74 ± 0.54^c^	8.28 ± 0.52^a^	20.42 ± 0.57^c^	27.11 ± 0.87^e^	15.14 ± 0.90^b^	19.49 ± 0.78^c^	24.69 ± 1.03^d^	15.39 ± 0.56^b^	0.013
VitC-B	17.30 ± 0.77^c^	6.59 ± 0.33^a^	17.02 ± 0.56^c^	24.48 ± 0.89^e^	10.68 ± 0.78^b^	16.60 ± 0.66^c^	21.95 ± 0.69^d^	9.06 ± 0.15^b^	0.018

Values in the same columns with different superscript (a, b, c, d, e, f) differ significantly. Data expressed as Mean ± SE (n = 6). GPx: Units/mg protein.; LPO: n mole TBARS formed/h/mg protein; Vit C: µg/g of wet tissue; AChE: nmole/min/mg protein.

### Zn-NPs on lipid peroxidation (LPO) and Vitamin C

Data on LPO in the liver and kidney and vitamin C in muscle and brain are recorded in Table 5. Zn-NPs at 4 mg kg^−1^ diet with or without stressors was noticeably reduces the LPO level in the liver (*p* = 0.0011) and gill (*p* = 0.0018) tissues compared to control and stressor group. However, the LPO level was significantly elevated with concurrent exposure to arsenic, ammonia and high temperature stress. Vitamin C in muscle (*p* = 0.013) and brain (*p* = 0.018) was noticeably reduced with As + T + NH_3_, compared to control and Zn-NPs supplemented groups. Further, supplementation of Zn-NPs at 4 mg kg^−1^ diet significantly elevated the Vit C in both the tissues (muscle and brain) with or without stressors compared to control and other groups. The other supplemented groups viz. Zn-NPs at 2 and 6 mg kg^−1^ diets did not affect the LPO and Vit C levels in fish.

### Zn-NPs and neurotransmitter enzymes

Acetylcholine esterase (AChE) in brain tissue was significantly inhibited (*p* = 0.0046) by concurrently exposed to As + T + NH_3_ in comparison to control and other supplemented groups. However, the AChE activities were significantly enhanced with supplementation of Zn-NPs at 4 mg kg^−1^ diet with or without stressors followed by Zn-NPs at 2 mg kg^−1^ diet group compared to control and other groups. In compared to Zn-NPs at 2 and 4 mg kg^−1^ diet, the Zn-NPs at 6 mg kg^−1^ diet was not substantially effective to enhances the AChE activities (Table 5).

### Zn-NPs and primary stress hormone and stress protein

**Figure 2:**
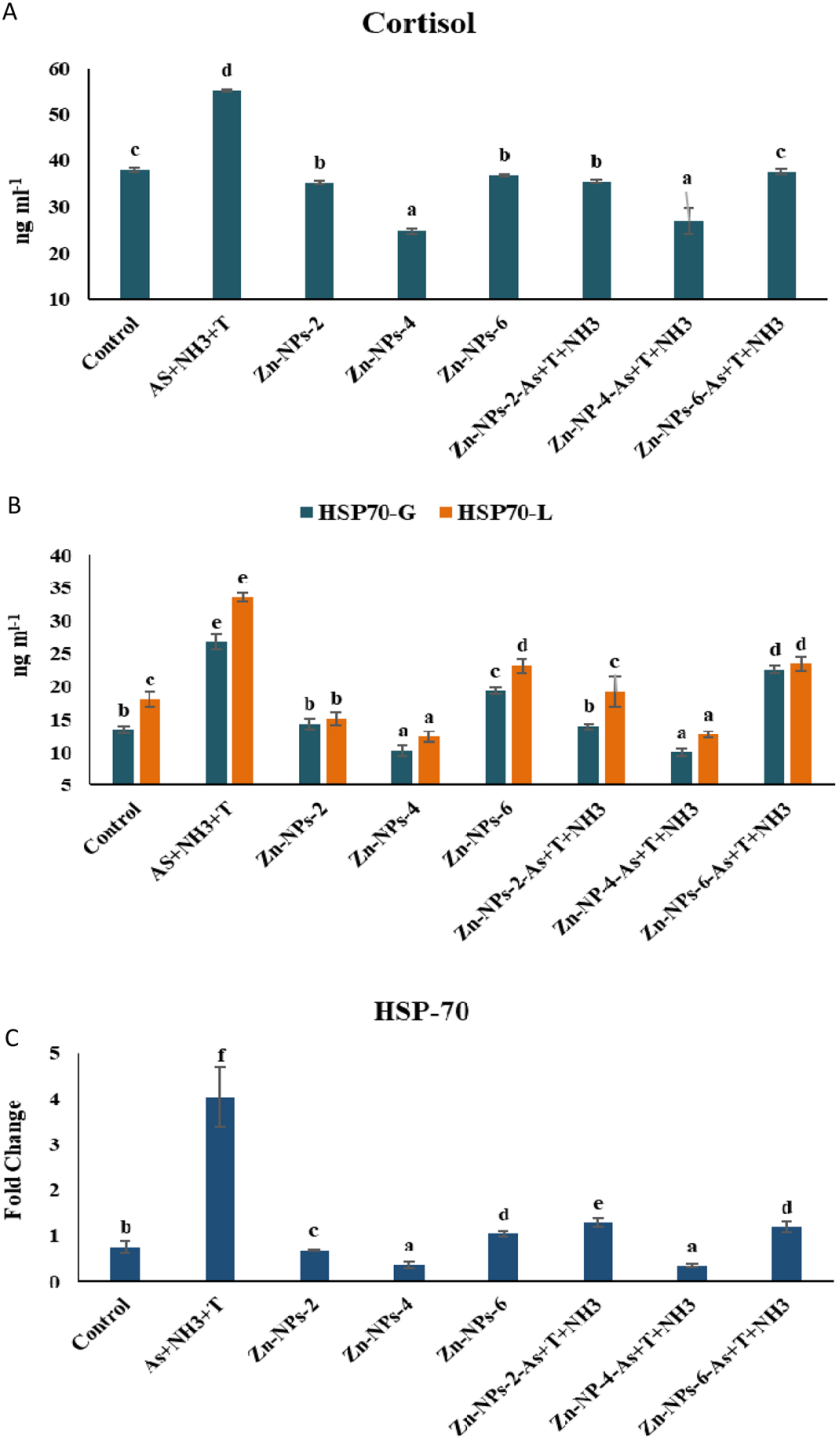
(**A**–**C**) Effect of dietary Zn-NPs on cortisol, HSP 70 in liver and gill and HSP 70 gene expression in muscle tissue of *P. hypophthalmus* reared under control condition or arsenic, ammonia and high temperature for 105 days. Within endpoints and groups, bars with different superscripts differ significantly (a–h) Cortisol (*p* = 0.0024), HSP-L (*p* = 0.0013), HSP-G (*p* = 0.0034) HSP 70-M (*p* = 0.0022). Data expressed as Mean ± SE (n = 3).

Cortisol was noticeably reduced (*p* = 0.0024) by supplementation of Zn-NPs at 4 mg kg^−1^ diet followed by the same feeding group, but concurrent exposure to As + T + NH_3_ compared to control and supplemented diets groups. Whereas, the cortisol was significantly higher with exposure to As + T + NH_3_. Moreover, Zn NPs at 2 and 6 mg kg^−1^ diets without stressors also significantly decreased the cortisol levels compared to the control and stressor group (Fig. 2A). Further, HSP 70 level was quantified using the kit and RNA expression using quantitative PCR. HSP level in gill was significantly reduced with supplementation of Zn-NPs at 4 mg kg^−1^ diet followed by 2 mg kg^−1^ diet in comparison to all other groups. Further, concurrent exposure to As + T + NH_3_ was significantly elevated the cortisol level (Fig. 2B). However, concerning fold change, the HSP 70 gene expression in muscle tissue was significantly (*p* = 0.0022) upregulated by As + T + NH_3_ in comparison to control and other groups. Moreover, the HSP 70 gene expression noticeably reduced the fold change in the group fed with Zn-NPs at 4 mg kg^−1^ followed by 2 mg kg^−1^ compared to control and other groups (Fig. 2C).

### Zn-NPs on iNOS, blood glucose, and DNA damage of the fish reared under multiple stresses

**Figure 3:**
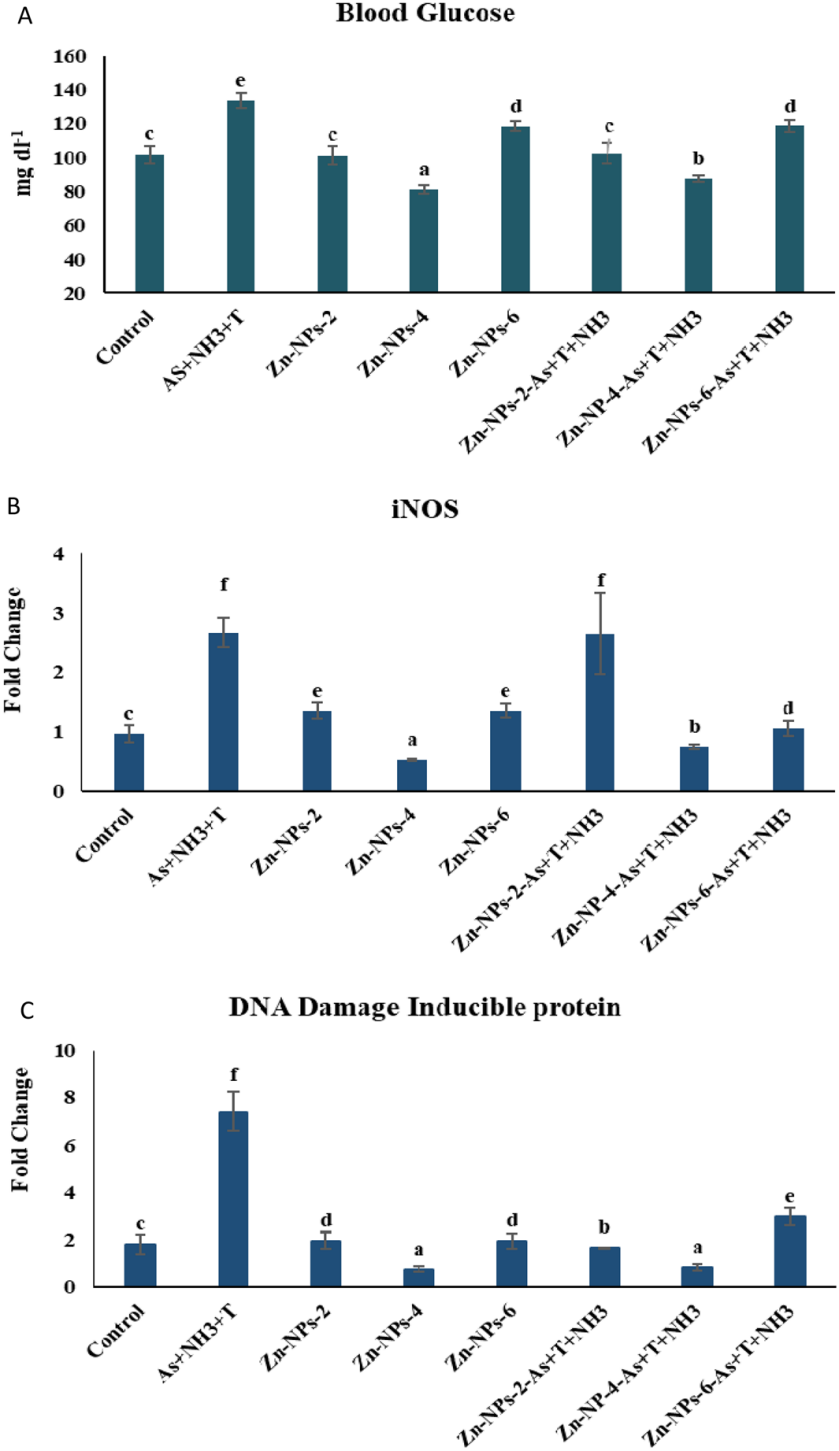
(**A**–**C**) Effect of dietary Zn-NPs on blood glucose (*p* = 0.011), iNOS (*p* = 0.0018) and DNA damage inducible protein (*p* = 0.0021) of *P. hypophthalmus* reared under control condition or arsenic, ammonia and high temperature for 105 days. Within endpoints and groups, bars with different superscripts differ significantly (a–g). Data expressed as Mean ± SE (n = 3).

Inducible nitric oxide synthase (iNOS) and DNA damage inducible protein in muscle and blood glucose are recorded in Fig. 3A–C. Concurrent exposure to arsenic, ammonia, and high temperature stress were noticeably upregulated the iNOS gene (*p* = 0.0018), DNA damage inducible protein (*p* = 0.0021) gene expressions and enhanced the blood glucose compared to control and other groups. Applying Zn-NPs at 4 mg kg^−1^ diet with or without stressors significantly downregulated the iNOS and DNA damage inducible protein and blood glucose compared to control and other groups.

### Zn-NPs on blood profiling of the fish reared under multiple stresses

**Figure 4:**
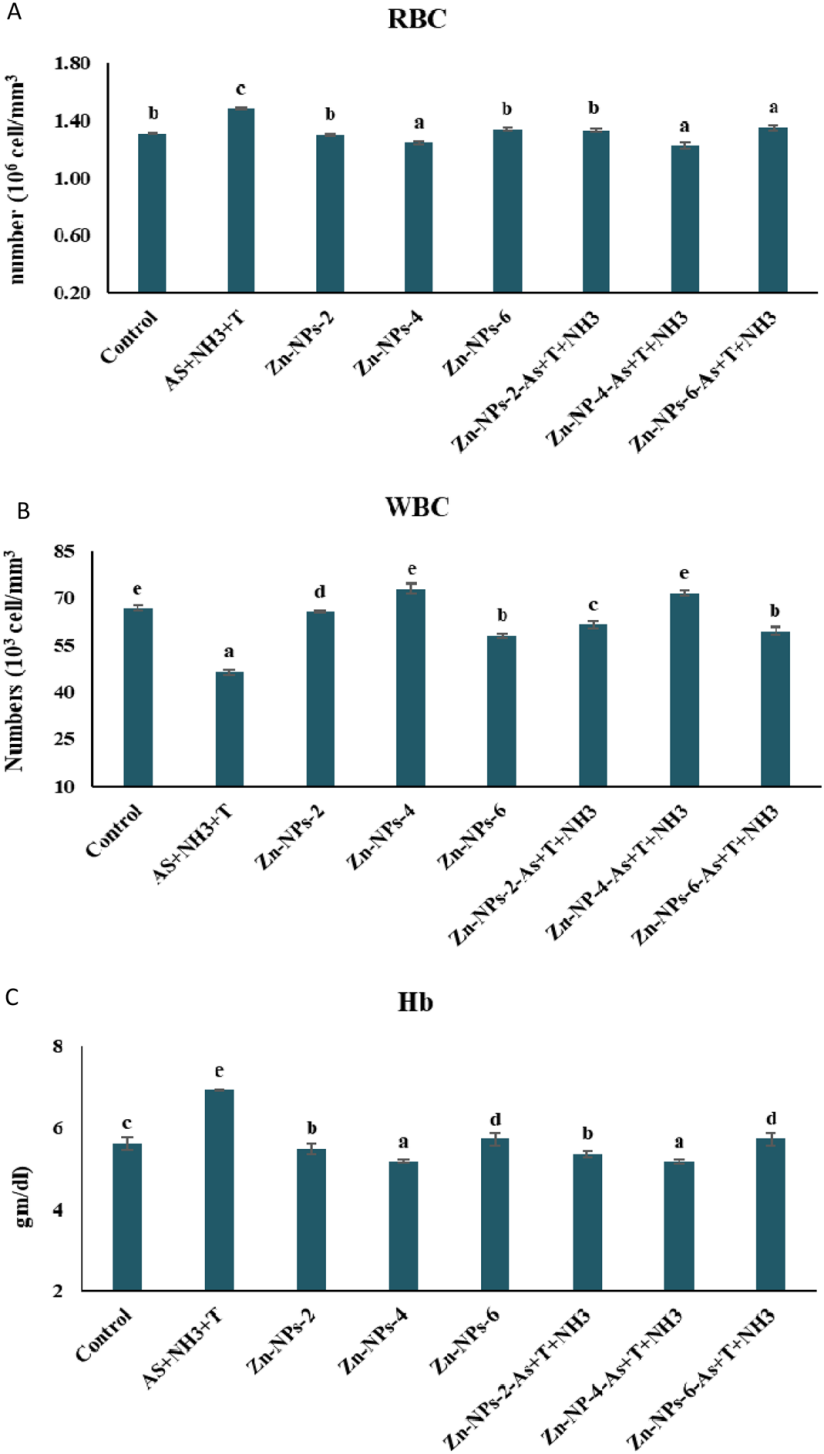
(**A**–**C**) Effect of dietary Zn-NPs on RBC, WBC and Hb of *P. hypophthalmus* reared under control condition or arsenic, ammonia and high temperature for 105 days. Within endpoints and groups, bars with different superscripts differ significantly, (a–f); RBC (*p* = 0.0022); WBC (*p* = 0.0015); Hb (*p* = 0.014). Data expressed as Mean ± SE (n = 3).

Blood profiling (RBC, WBC and Hb) of the fish reared under different stress condition as arsenic, ammonia and high temperature for 105 days. RBC (*p* = 0.0022), WBC (*p* = 0.0015) and Hb (*p* = 0.014) were remarkably reduced with As + T + NH_3_ in comparison to control and other groups. Moreover, the RBC, WBC and Hb were significantly enhanced with dietary Zn-NPs at 4 mg kg^−1^ compared to the control and other groups (Fig. 4A–C). The results of blood glucose also showed the Zn-NPs at 6 mg kg^−1^ diet was not effective to modulate the blood count of the fish with or without exposure to stresses.

### Zn-NPs on the immunity of the fish

Data about immune-related attributes (Total protein, globulin, A:G ratio, nitroblue tetrazolium (NBT), immunoglobulin, myeloperoxidase) are recorded in Table 5. The supplementation of Zn-NPs at 4 mg kg^−1^ diet significantly improved the total protein (*p* = 0.015) and globulin (*p* = 0.012) and reduced the A:G ratio (*p* = 0.018) compared to all other groups in *P. hypophthalmus* compared to control and other groups. NBT was significantly suppressed (*p* = 0.014) by As + T + NH_3_ in comparison to control and other groups. Whereas, NBT was noticeably enhanced with dietary Zn-NPs at 4 mg kg^−1^ diet compared to control and other groups. Similarly, immunoglobulin (Ig) (*p* = 0.0062) and myeloperoxidase (MPO) (*p* = 0.0036) were noticeably enhanced with Zn-NPs at 4 mg kg^−1^ diet in comparison to all other groups. Ig and MPO were significantly reduced with concurrent exposure to As + T + NH_3_ in comparison to all the groups.  Moreover, Zn-NPs at 6 mg kg^−1^ diet fed group was not effective compared to Zn-NPs at 2 and 4 mg kg^−1^ diet (Table 6).

**Table 6:** Effect of dietary Zn-NPs on immunity index (total protein, albumin, globulin, A:G ration, NBT, Ig and MPO) of *P. hypophthalmus* reared under control condition or arsenic, ammonia and high temperature stress for 105 days.

Treatments	Non-Stressors	Stressors (As + NH_3_ + T)	Non-Stressors			Stressors (As + NH_3_ + T)			*P*-Value
Diets	Control	Control	Zn-NPs-2	Zn-NPs-4	Zn-NPs-6	Zn-NPs-2	Zn-NPs-4	Zn-NPs-6	
Total Protein	0.63 ± 0.02^d^	0.27 ± 0.02^a^	0.67 ± 0.05^d^	0.88 ± 0.04^e^	0.38 ± 0.02^b^	0.64 ± 0.07^d^	0.85 ± 0.03^e^	0.50 ± 0.02^c^	0.015
Globulin	0.38 ± 0.01^c^	0.16 ± 0.01^a^	0.49 ± 0.03^de^	0.74 ± 0.06^f^	0.26 ± 0.02^b^	0.41 ± 0.03^d^	0.68 ± 0.03^e^	0.35 ± 0.02^c^	0.012
A:G ratio	0.69 ± 0.04^g^	0.70 ± 0.03^f^	0.37 ± 0.03^c^	0.19 ± 0.01^a^	0.51 ± 0.04^d^	0.60 ± 0.04^e^	0.26 ± 0.03^b^	0.42 ± 0.02^cd^	0.018
NBT	0.57 ± 0.05^c^	0.36 ± 0.02^a^	0.58 ± 0.02^c^	0.67 ± 0.04^d^	0.43 ± 0.02^b^	0.54 ± 0.07^d^	0.64 ± 0.03^d^	0.44 ± 0.02^b^	0.0047
Ig	0.40 ± 0.02^c^	0.15 ± 0.01^a^	0.51 ± 0.03^d^	0.68 ± 0.04^e^	0.17 ± 0.01^a^	0.28 ± 0.02^b^	0.64 ± 0.05^e^	0.30 ± 0.02^b^	0.0062
MPO	0.29 ± 0.02^c^	0.16 ± 0.01^a^	0.32 ± 0.02^c^	0.38 ± 0.03^d^	0.20 ± 0.01^b^	0.28 ± 0.02^c^	0.38 ± 0.02^d^	0.22 ± 0.01^b^	0.0036

Values in the same columns with different superscript (a, b, c, d, e) differ significantly. Total protein, albumin, globulin: g dL^−1^Blood glucose: mgdL^−1^; Data expressed as Mean ± SE (n = 3).

### Zn-NPs on the immune gene of the fish

**Figure 5:**
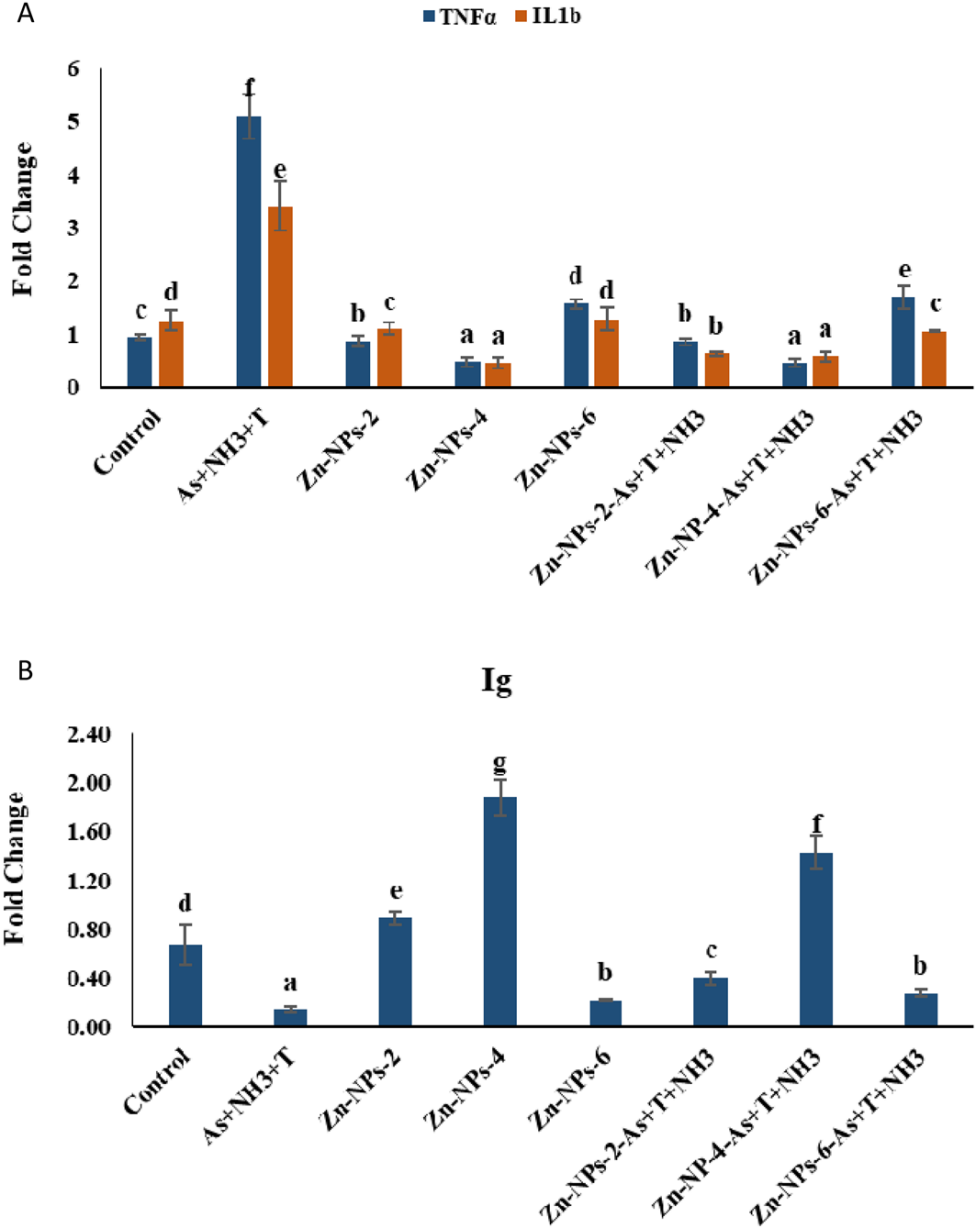
(**A**,**B**) Effect of dietary Zn-NPs on tumour necrosis factor (TNFα), interleukin (IL1b) and immunoglobulin (Ig) of *P. hypophthalmus* reared under control condition or arsenic, ammonia and high temperature for 105 days. Within endpoints and groups, bars with different superscripts differ significantly, (a–h); TNFα (*p* = 0.0017); IL1b (*p* = 0.029); Ig (*p* = 0.0034). Data expressed as Mean ± SE (n = 3).

In the present investigation, tumor necrosis factors (TNFα), interleukin (IL1β), and immunoglobulin gene expressions were quantified and shown in Fig. 5A,B. The immune-related genes TNFα (*p* = 0.0017), and IL1β (*p* = 0.0029) were noticeably reduced with Zn-NPs at 4 mg kg^−1^ diet in comparison to control and other groups. Interestingly, TNFα and IL1β were significantly enhanced with As + T + NH_3,_ in comparison to control and other groups. Further, Ig gene was noticeably enhanced (*p* = 0.0034) with supplementation of Zn-NPs at 4 mg kg^−1^ diet with or without stressors (As + T + NH_3_) followed by Zn-NPs at 2 mg kg^−1^ diet in comparison to all other groups. Surprisingly, the concurrent exposure to As + NH_3_ + T downregulated the Ig gene in muscle tissue of *P hypophthalmus*.

### Zn-NPs on the genotoxicity of the fish

Genotoxicity viz. comet assay for DNA damage was determined in kidney tissue of the fish reared under As + NH_3_ + T. The data are recorded in Table 7. Tail DNA % was noticeably reduced with Zn-NPs at 4 mg kg^−1^ (5.50%) diet, followed by control group (10.40%) and Zn-NPs at 2 mg kg^−1^ (15.30%) diet without stressors. The tail DNA % was observed highest in group exposed to As + T + NH_3_ (89.36%). In the case of Zn-NPs at 4 mg kg^−1^ diet with stressors, the tail DNA % was 18.11%. Among the Zn-NPs treated groups, the highest DNA damage was observed in the group treated with Zn-NPs at 6 mg kg^−1^ diet with or without stressors.

**Table 7:** Effect of dietary Zn-NPs on genotoxicity (DNA damage) in kidney tissue of *P. hypophthalmus* reared under control condition or arsenic, ammonia and high temperature stress for 105 days.

Treatments	Non-Stressors	Stressors (As + NH_3_ + T)	Non-Stressors			Stressors (As + NH_3_ + T)		
Diets	Control	Control	Zn-NPs-2	Zn-NPs-4	Zn-NPs-6	Zn-NPs-2	Zn-NPs-4	Zn-NPs-6
Comet Length	20 ± 0.65	18 ± 0.82	29 ± 0.07	28 ± 0.081	29 ± 0.02	68 ± 0.08	25 ± 0.12	30 ± 0.17
Comet DNA	1366 ± 22.13	5005 ± 11.26	8092 ± 18.23	5659 ± 19.65	98,792 ± 21.94	25,451 ± 72.98	4891 ± 34.22	8688 ± 24.39
Head Area	401 ± 11.29	4 ± 0.02	463 ± 11.53	582 ± 15.28	48 ± 1.67	80 ± 3.41	469 ± 7.46	4 ± 0.13
Head DNA	1224 ± 17.73	3 ± 0.13	6710 ± 16.29	5348 ± 11.23	671 ± 1.29	617 ± 4.53	4005 ± 11.27	25 ± 0.89
Head DNA (%)	89.60 ± 3.98	10.64 ± 0.91	84.70 ± 0.05	94.50 ± 3.28	69.79 ± 1.27	60.56 ± 2.67	81.89 ± 0.98	40.86 ± 2.12
Tail DNA	142 ± 6.29	5002 ± 11.22	1382 ± 10.83	311 ± 9.21	98,121 ± 67.62	24,834 ± 18.67	886 ± 5.28	8663 ± 17.78
Tail DNA (%)	10.40 ± 0.87	89.36 ± 2.76	15.30 ± 1.14	5.50 ± 0.38	30.21 ± 1.35	39.44 ± 1.51	18.11 ± 1.12	59.14 ± 2.76

Data expressed as Mean ± SE (n = 3).

### Zn-NPs on growth performance of the fish

The growth performance such as final body weight gain (%), feed conversion ratio (FCR), specific growth rate (SGR), protein efficiency ratio (PER), daily growth index (DGI %), thermal growth coefficient (TGC) and relative feed intake (RFI) were determined in the fish reread under As + NH_3_ + T and fed with Zn-NPs diets (Table 8). In the present investigation, the body weight gain % was significantly enhanced (*p* = 0.0012) with supplementation of Zn-NPs at 4 mg kg^−1^ diet with or without stressors compared to control and other groups. Interestingly, the growth was drastically reduced with concurrent exposure to stressors (As + NH_3_ + T) compared to the control and supplemented group. Further, SGR (*p* = 0.0017) and PER (*p* = 0.0043) were remarkably reduced with As + NH_3_ + T. In contrast, the SGR, PER, DGI and RFI were significantly enhanced with supplementation of Zn-NPs at 4 mg kg^−1^ diet compared to the control and other groups. Moreover, the results of FCR were inverse of SGR and PER, as FCR was significantly enhanced (*p* = 0.0012) with stressors and lowest with Zn-NPs at 4 mg kg^−1^ diet supplementation. Moreover, the TGC was non-significant among all the groups (*p* > 0.05).

**Table 8:** Effect of dietary Zn-NPs on growth performance (final body weight gain %, FCR, SGR, PER, DGI, TGC and RFI) of *P. hypophthalmus* reared under control condition or arsenic, ammonia and high temperature stress for 105 days.

Treatments	Non-Stressors	Stressors (As + NH_3_ + T)	Non-Stressors			Stressors (As + NH_3_ + T)			*P*-Value
Diets	Control	Control	Zn-NPs-2	Zn-NPs-4	Zn-NPs-6	Zn-NPs-2	Zn-NPs-4	Zn-NPs-6	
Final body weight gain %	140.86 ± 9.86^c^	75.89 ± 2.34^a^	168.12 ± 9.21^d^	246.42 ± 2.12^e^	108.07 ± 2.86^b^	169.34 ± 2.01^d^	259.05 ± 10.04^e^	119.40 ± 6.03^b^	0.0012
FCR	2.43 ± 0.11^c^	3.80 ± 0.08^d^	2.13 ± 0.09^b^	1.72 ± 0.06^a^	3.09 ± 0.12^cd^	2.15 ± 0.03^b^	1.63 ± 0.17^a^	2.83 ± 0.14^c^	0.0012
SGR	0.80 ± 0.05^b^	0.52 ± 0.05^a^	0.92 ± 0.08^c^	1.20 ± 0.07^d^	0.76 ± 0.02^b^	0.96 ± 0.06^d^	1.20 ± 0.11^d^	0.85 ± 0.09^bc^	0.0017
PER	1.19 ± 0.09^c^	0.82 ± 0.02^a^	1.47 ± 0.08^de^	1.88 ± 0.21^e^	0.93 ± 0.04^b^	1.37 ± 0.03^d^	1.81 ± 0.09^e^	1.12 ± 0.07^c^	0.0043
DGI (%)	1.24 ± 0.05^c^	0.73 ± 0.02^a^	1.47 ± 0.06^d^	1.92 ± 0.01^e^	1.02 ± 0.02^b^	1.48 ± 0.01^d^	2.04 ± 0.04^e^	1.11 ± 0.05^b^	0.012
TGC	0.0396	0.0306	0.0391	0.0382	0.04	0.0305	0.0308	0.0308	0.094
RFI	340.86 ± 9.75^b^	288.20 ± 2.96^a^	355.82 ± 4.31^c^	424.34 ± 2.48^e^	333.85 ± 1.61^b^	363.47 ± 2.14^d^	421.63 ± 6.64^e^	336.55 ± 2.75^b^	0.017

Values in the same columns with different superscript (a, b, c, d, e) differ significantly. Data expressed as Mean ± SE (n = 3).

*FCR* feed conversion ratio, *SGR* specific growth rate, *PER* protein efficiency ratio, *DGI* Daily growth index, *TGC* Thermal growth coefficient, *RFI* relative feed intake.

### Zn-NPs on genes related to the growth performance of the fish

**Figure 6:**
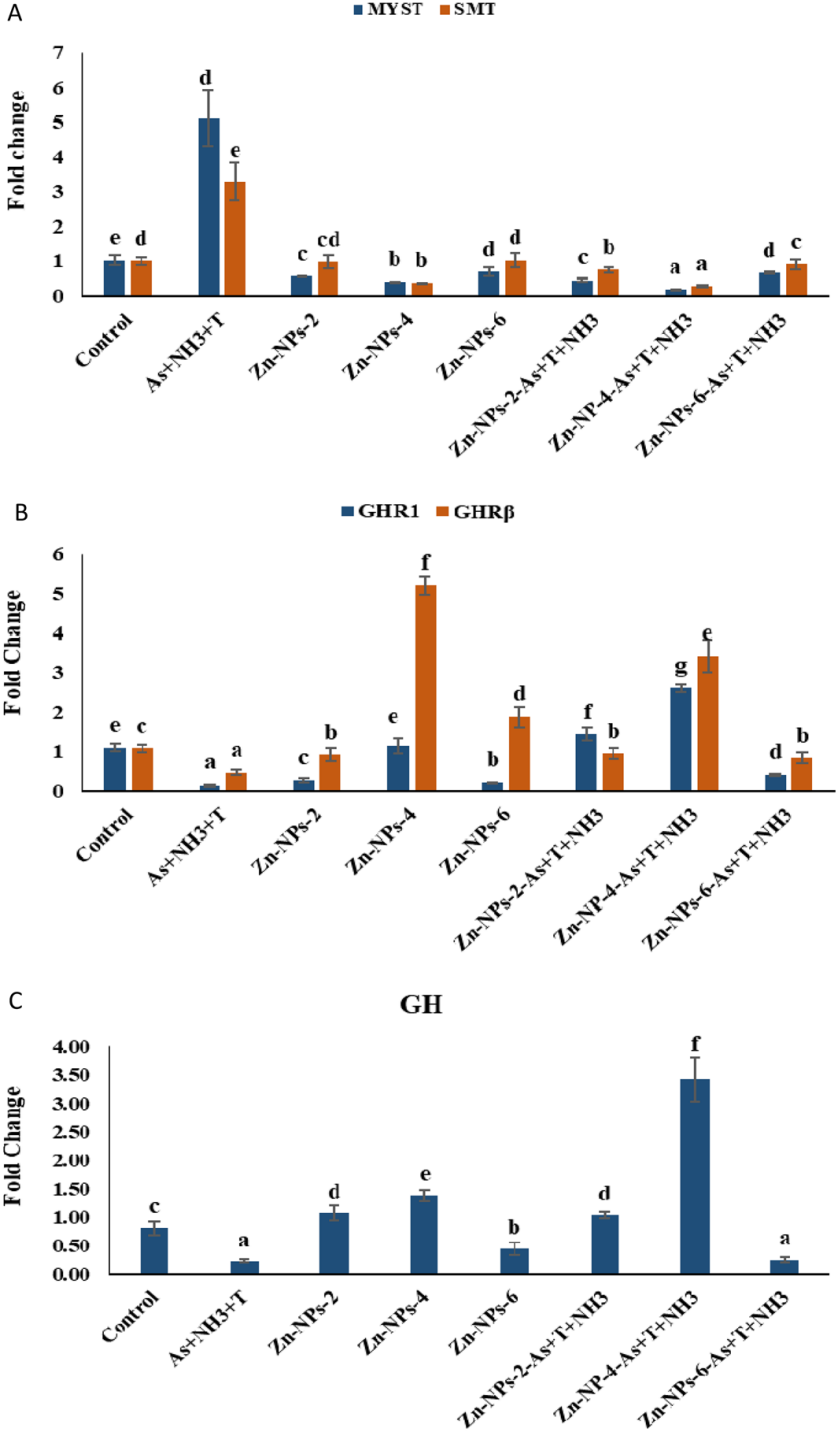
(**A**–**C**) Effect of dietary Zn-NPs on myostatin, somatostatin, growth hormone regulator1 and β and growth hormone gene quantification of *P. hypophthalmus* reared under control condition or arsenic, ammonia and high temperature for 105 days. Within endpoints and groups, bars with different superscripts differ significantly, (a–h); MYST (*p* = 0.011); SMT (*p* = 0.018); GHR1 (*p* = 0.0023); GHRβ (*p* = 0.0014) and GH (*p* = 0.0031). Data expressed as Mean ± SE (n = 3).

Gene regulation and expression of growth hormone (GH), growth regulatory hormone (GHR1 and GHRβ), myostatin (MYST), and somatostatin (SMT) in muscle tissue of *P. hypophthalmus* reared under concurrent exposure to arsenic, ammonia and high temperature stress. The data are shown in Fig. 6A–C. GH (*p* = 0.0031), GHR1 (*p* = 0.0023) and GHRβ (*p* = 0.014) genes were noticeably upregulated by supplementation of Zn-NPs at 4 mg kg^−1^ diet with or without stressors in comparison to control and other groups. The group exposed to As + NH_3_ + T significantly downregulated the GH, GHR1 and GHRβ genes compared to control and other supplemented groups. Further, the MYST (*p* = 0.011) and SMT (*p* = 0.018) genes were remarkably downregulated with supplementation of Zn-NPs at 4, 2, and 6 mg kg^−1^ diet. Moreover, MYST and SMT gene regulation were significantly upregulated with the stressors group compared to the control and other groups.

### Arsenic concentration and bioaccumulation in water and fish tissues

Data on arsenic concentration in water and bioaccumulation in the liver, muscle, gill, kidney and brain tissues of *P. hypophthalamus* is recorded in Table 9. The highest concentration of arsenic was determined in the group treated with As + NH_3_ + T. Interestingly, supplemented group of Zn-NPs at 6 and 2 mg kg^−1^ diets with stressors had higher arsenic concentrations. Surprisingly, higher arsenic bioaccumulation was determined in the liver and kidney tissues. The highest As bioaccumulation was determined in the group As + NH_3_ + T. The supplementation of Zn-NPs at 4 mg kg^−1^ diet did not detect arsenic bioaccumulation in non-exposed conditions.

**Table 9:** Effect of dietary Zn-NPs on arsenic bioaccumulation in water and different fish tissues of *P. hypophthalmus* reared under control condition or arsenic, ammonia and high temperature stress for 105 days.

Treatments	Non-Stressors	Stressors (As + NH_3_ + T)	Non-Stressors			Stressors (As + NH_3_ + T)		
Diets	Control	Control	Zn-NPs-2	Zn-NPs-4	Zn-NPs-6	Zn-NPs-2	Zn-NPs-4	Zn-NPs-6
Water (µg L^−1^)	0.07 ± 0.01	1657.95 ± 68.76	0.07 ± 0.01	0.07 ± 0.01	0.07 ± 0.00	1034.27 ± 48.05	433.79 ± 11.09	1196.31 ± 114.18
Liver	0.02 ± 0.00	6.37 ± 0.06	0.01 ± 0.0	ND	0.01 ± 0.0	2.00 ± 0.17	0.25 ± 0.05	2.05 ± 0.04
Muscle	ND	1.74 ± 0.21	ND	ND	0.03 ± 0.01	1.85 ± 0.02	0.33 ± 0.01	1.17 ± 0.05
Gill	0.01 ± 0.0	1.76 ± 0.03	0.03 ± 0.0	0.02 ± 0.0	0.07 ± 0.02	2.12 ± 0.12	0.32 ± 0.12	0.01 ± 0.08
Kidney	0.03 ± 0.0	4.37 ± 0.11	0.03 ± 0.01	0.02 ± 0.01	0.21 ± 0.05	2.61 ± 0.08	0.19 ± 0.03	0.93 ± 0.14
Brain	ND	0.37 ± 0.02	ND	ND	0.04 ± 0.0	0.80 ± 0.01	0.06 ± 0.0	0.88 ± 0.02

Data expressed as Mean ± SE (n = 3).

### Zinc concentration in diets and muscle tissue

**Figure 7:**
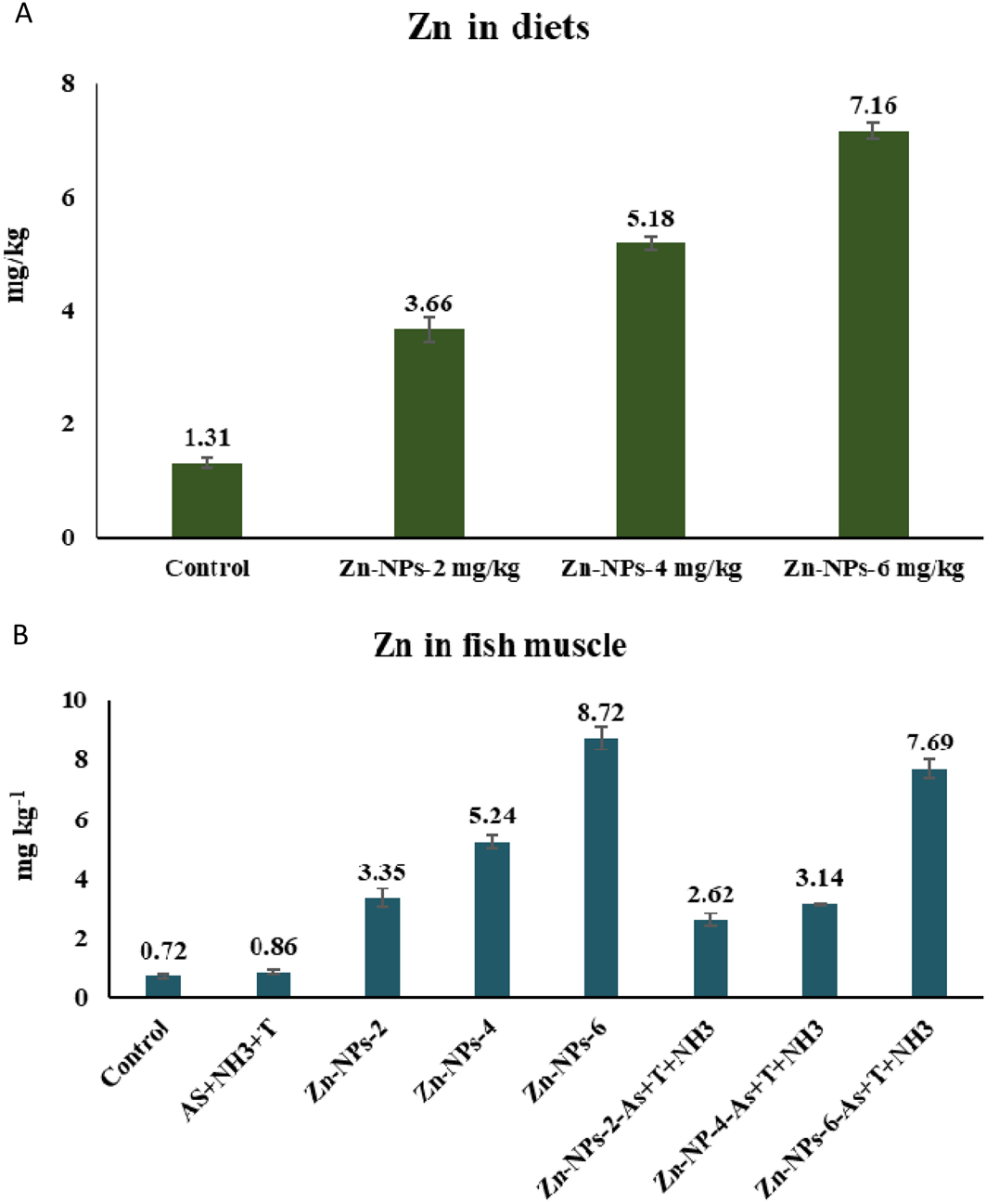
(**A**,**B**) Zinc content in diets (**A**) and muscle tissue (**B**).

Zinc concentration in fish diets viz. control, Zn-NPs at 2, 4 and 6 mg kg^−1^ were determined as 1.3, 3.16, 5.18 and 7.16 mg kg^−1^ diets. Zn bioaccumulation in muscle was determined as 0.72, 0.86, 3.35, 5.24, 8.72, 2.62, 3.14, and 7.69 mg kg^−1^ tissue in control, As + NH_3_ + T, Zn-NPs at 2, 4 and 6 mg kg^−1^ diet without stressors and with stressors (Fig. 7A,B).

## Discussion

The present investigation addresses ammonia, arsenic toxicity and high temperature stress, which affects on sturdy species *P. hypophthalmus* rearing in adverse conditions. This study focused on the impact of arsenic, ammonia and high temperature (34 °C) (As + NH_3_ + T) stress on gene regulations in different physiological and molecular response, whereas mitigation of such stresses through dietary Zn-NPs. Moreover, the concurrent exposure to As + NH_3_ + T enhanced the toxicity and affected the anti-oxidative status of the fish. Concurrent exposure to As, NH_3_ and T enhanced the SOD, CAT, GST and GPx activities in the liver, kidney and gill tissues. Ammonia toxicity can also induce the overproduction of reactive oxygen species (ROS), damaging the protein, lipid and DNA^51^. This process initiates the cascade events, which result in impaired fish cellular and physiological function^52,53^. Moreover, excess ROS production damages and impairs the respiratory chain and mitochondrial membrane permeability. Further, under stress-free conditions, the anti-oxidant defense system quickly removed the ROS^54,55^. Therefore, the activities of CAT, SOD, GST and GPx were elevated with stressor As + NH_3_ + T in the liver, kidney and gill tissues of the fish. Our previous study reported that temperature stress enhances arsenic toxicity in aquatic animals^56,57^. Arsenite (III) and arsenate (V) are the inorganic form of arsenic, which induce the redox system to release or generate ROS^58^. Moreover, arsenic produces free radicals in two ways: redox active metals and without redox potential. Interestingly, the transcription factors viz. p-53, NF-κB and AP-1 also help in the generation of free radicals, which stop the DNA repairs and control the protective gene expressions as well as cell growth and differentiation^59,60^. In this investigation, Zn-NPs showed mitigating abilities of stresses such as arsenic, ammonia, and high temperature (34 °C). Zinc is vital in anti-oxidant as it acts as a co-factor for more than 300 enzymes and plays an important role in cell division, growth and genetic expression^61^. It is also an integral parts of the Cu–Zn-SOD (superoxide dismutase) complex, which indicates that it has scavenging properties. Interestingly, SOD has three isoforms which are characterised by cellular localization and metal co-factors such as Zn-Cu-SOD. It is present in the cytosol as SOD-1, second is Mn dependent SOD located in mitochondria (Mn-SOD) called as SOD-2 and last one extracellular Cu/Zn SOD, located within the extracellular matrices of tissues (EC-SOD; SOD-3)^62,63^. SOD is also playing crucial roles for development and maintenance of a homeostatic balance between the innate immune response and antioxidant defenses^64^. Moreover, this mechanism was applied by the Zn-NPs for mitigation of multiple stressors. In the present investigation, to cope up with excessive generation of reactive oxygen species (ROS) due to multiple stresses (Arsenic, ammonia and high temperature), a cellular detoxification system works using antioxidants and non-enzymatic small molecules such as CAT, SOD, GPx and GST^65^. Surprisingly, CAT have an important role as oxidoreductase enzyme that converts H_2_O_2_ to H_2_O and O_2_. Moreover, at low level of H_2_O_2_ it can oxidize the toxins viz. phenols, alcohol, formic acid, and formaldehyde using one molecule of H_2_O via peroxidatic pathway^66^. In continuation with above mechanism, Zn-NPs also participated in the scavenging properties and inhabitation of NADPH-oxidase enzyme (nicotinamide adenine dinucleotide phosphate oxidase), stabilizing the cell membranes and induced metallothionein synthesis^67,68^. NADPH-Oxidase enzymes (nicotinamide adenine dinucleotide phosphate oxidase) are multi-subunit protein complexes that majorly transfer electrons across the plasma membrane to molecular oxygen. This function generates the superoxide anion and ROS, including hydrogen peroxide (H_2_O_2_) and hydroxyl radicals (OH^−^)^69^. Moreover, it also enhances the activities of reduced glutathione (GSH) activities, which has an important role against oxidative stress^70^. Zn is also important for synthesizing cysteine-rich, metal-binding metallothionein protein, which enhances the scavenging of free radicals^71^. Our earlier study also demonstrated that Zn and Zn-NPs reduce the toxicity of lead and high temperature^20,21^. Our earlier study also reported that Se-NPs has important role in mitigation of multiple stressors (arsenic toxicity and high temperature stress) in fish. Se-NPs showed anti-oxidative property mainly due to selenoprotein synthesis that is depending upon the glutathione peroxidase (GPx). Moreover, it has also limitation for stress mitigation because, the small change in concentration of Se can induced the toxicity and produces free radicals inside the cell^16,22^. The present investigation is the first to report the mechanistic role of Zn-NPs in mitigating multiple stresses (As + NH_3_ + T) with special reference to NFkB regulation, genotoxicity and detoxification mechanism in *P. hypophthalmus*.

Lipid peroxidation (LPO) plays a vital role during oxidative stress, which induces the loss of cell function^72^. In the present investigation, the ammonia, arsenic toxicity and high temperature stress cause the LPO in different fish tissues. It could be due to these abiotic factors induced uncontrolled oxidative damage, as reflected in this study^73^. Excessive ROS production is directly related to LPO as destroys the cell membranes due to alteration in LPO level. However, arsenic also produces ROS using three sources such as (a) it reacts with acid and forms arsine^74^, (b) ferritin forms methylated redox-active iron, which generates ROS using O_2_^−^ and H_2_O_2_ into the highly reactive ^−^OH radical^75^ and (c) ROS produce during conversion of arsenite to arsenate^76^. Zn-NPs protect the cell against oxidative damage by reducing lipid peroxidation, enhancing Vit C, and minimizing cell damage^77^. Zn also takes vital role in the Cu–Zn-SOD complex. Hence, managed the oxidative stress, LPO and Vit C level in the cell^78^.

AChE activities were noticeably reduced with exposure to ammonia, arsenic and high temperature stress might be due to hyperactivation of its receptors and accumulation of NH_3_. The toxicity of NH_3_, arsenic and high temperature stress also induces the breakdown of acetylcholine by acetylcholinesterase which also affects synthesis and release of the neurotransmitter. Moreover, supplementation of dietary Zn-NPs at 4 mg kg^−1^ diet enhanced the AChE activities in brain tissue due to its role in exocytosis and formation of synaptic vesicles containing neurotransmitters^21^.

In the present investigation, the HSP70 and cortisol were elevated due to ammonia, arsenic and high temperature stress. HSPs are the stress chaperone protein that protects the cell against oxidative stress via preventing protein aggregation, refolding and/or folding of nascent protein^79,80^. It also involves antigen processing, stress response, anti-apoptotic and intracellular trafficking^81^. Moreover, As + NH_3_ + T has altered the protein structure as protein aggregation, denaturation and cortisol levels. The alteration in cortisol secretion comprises fish health as it is involved in metabolism, immunity, and secretion^82^. Indeed, the supplementation of Zn-NPs significantly reduces the effect of stressors and decreases the HSP 70 and cortisol in both tissues might be due to its role in the adrenal gland and adrenocorticotropic hormone regulation using the blood–brain barrier^83^. Zn also modulates the HSP using the folding of newly synthesized polypeptides and provides a chaperone environment for translation. However, this is the mechanism involved in modulating HSP 70 by Zn-NPs^84,85^.

Generally, ammonia is accumulated in different tissues and produces nitric oxide. However, ammonia is one of the factors influencing iNOS gene expression in different tissues including muscle^86^. Apart from ammonia, the arsenic and high temperature stress also induce the gene expression of the iNOS. Exposure of As + NH_3_ + T, the iNOS gene upregulated, resulting in tissue injuries, neurotoxicity and lipid oxidation^87^. Surprisingly, dietary Zn-NPs downregulate the iNOS gene expression in muscle tissue might be due to its role in NOS enzymes inhibiting catalytical activity^88^. Zinc down-regulated the expression of iNOS (mRNA + protein) and decreased cytokine-mediated activation of the iNOS promoter. Zinc-mediated regulation of iNOS expression inhibited the NF-κB transactivation activity, as determined by a decrease in NF-κB-driven luciferase reporter activity and expression of NF-κB target genes, including cyclooxygenase 2 and IL-1β^89^. It is also revealed that iNOS is also involved in zinc homeostasis and avoids oxidative stress^90,91^. This mechanism is involved in controlling and regulating the iNOS gene using Zn-NPs.

The blood glucose was elevated with exposure to arsenic, ammonia and high temperature, which might be due to role of gluconeogenetic and catecholamines involved in glycogenolytic activity under these stress condition^92^. Moreover, glucose passes through the cell and activates lipogenesis and glycogenesis. In the present investigation, concurrent exposure to As + NH_3_ + T, the stimulation receptor, communicated to hypothalmus and secreted catecholamine hormone, which allows glucose into the cell. In the case of the hyperglycaemic condition, it inhibited the glycogen formation and stopped the entry of glucose into the cell resulting in higher glucose levels^93^. However, in the present study, Zn-NPs were noticeably reducing blood glucose due to their role in glucose metabolism. It also reduces synthesis and absorption and, at the same time, enhances glucose metabolism and storage. It might be due to key enzymes involved during metabolisms, such as glycogen synthase, α-glucosidase, pyruvate kinase and phosphofructokinase. Further, Zinc-α2-glycoporteins mediated the insulinomimetic action, which enhanced the cellular GLUT4 levels in muscles and fatty tissue and facilitated glucose absorption^94^.

The toxic substances such as ammonia, arsenic, and high temperature altered the blood profiling (RBC, WBC, and Hb). The blood attributes are very sensitive and reliable indicator for evaluating aquatic animal health^95^. In the present study, the RBC, WBC and Hb were substantially reduced with exposure to As, NH_3_ and T stress, whereas supplementation of Zn-NPs enhanced the blood count in fish. Haemoglobin (Hb) levels were decreased with exposure to As, NH_3_ and T, possibly due to enhanced oxygen demand during exposure and hence elevation in methemoglobin by gill damage^96^. Interestingly, RBC and WBC were reduced with exposure to stress which might be due to anemia and leucopenia, that inhibits the erythropoietic process^97^. Indeed, supplementing Zn-NPs enhances fish’s total blood count (RBC, WBC and Hb). Zinc nanoparticles stimulate the erythropoiesis to maintain the RBC, WBC and Hb in the fish reared under As + NH_3_ + T.

Total protein, globulin, A:G ratio, Nitro blue tetrazolium chloride (NBT), total immunoglobulin and myeloperoxidase (MPO) are a reliable indicator for immunity. In the present study, innate immunity was altered by exposure to arsenic, ammonia and high-temperature stress in fish, whereas immunological attributes were improved by supplementation of Zn-NPs. The higher protein level, globulin and lower albumin indicates strong immunity in fish^98^. The lowest A:G ratio was determined in the Zn-NPs at 4 mg kg^−1^ diet supplemented group. Respiratory burst activity of phagocytes was determined using NBT which deals with superoxide radicals produced through leucocytes. Our earlier studies also reported similar results when fish were exposed to multiple stresses and fed with Zn and Zn-NPs^20,21^. Moreover, innate immunity plays an important role in maintaining integrity using antimicrobial molecules and phagocytosis. Albumin is also important for transporting biological molecules such as bilirubin, hormone, drug and vitamin. It is also involved in the regulation of hormones and fat metabolism^99^. In the present investigation, the immunoglobin (Ig) gene expression was determined as an adaptive immune response^100^. Ig has an important role in killing the various pathogen and microbes, restricting the dispersal of infectious agents, repairing and maintenance of tissue damage, and the health status of aquatic animals^101^. MPO is an important component for the immunity of the fish, which use during respiratory burst to form hydrogen peroxide to produce hypochlorous acid^102^. Hypochlorous acid is an oxidant that has a role in cytotoxic effects on bacterial and mammalian cells^103^. Moreover, the Zn-NPs improve the immunity of the fish using strengthening the immune-related attributes, viz. total protein, albumin, globulin, A:G ratio, NBT, Ig and MPO. It is also crucial for developing and functioning innate immunity-mediated cells NK cells and neutrophils. It also helps in intracellular killing, cytokine production, phagocytosis production, and growth and function of T and B cells^104^. The present study also deals with the gene expression of TNFα (Tumor necrosis factor) and IL1β (interferon) in response to ammonia, arsenic and high temperature stress in fish. Exposure to stressors, the TNFα and IL1β were noticeably upregulated in fish muscle tissue. TNFα and IL1β play an important role in regulating innate immune systems against infection and other stress condition^105^. Similarly, in this study, the ammonia, arsenic and high temperature exposure upregulated the TNFα and IL1b. TNFa is associated with B-cell activating factor (BAFF), which is directly involved in the NF-kB regulation. Moreover, the Zn-NPs activate the NF-κB signaling pathway, which enhances the immunity of fish against As + NH_3_ + T stress^106^. Arsenic, ammonia and high temperature stress induce DNA damage in kidney tissue and gene expression of DNA damage inducible protein in muscle tissue of the fish. It is well known that contamination such as arsenic and ammonia could induce genotoxicity in fish. In the present investigation, the arsenic, ammonia and high temperature stress cause oxidative stress, which is the primary process for genotoxicity as DNA damage. In this process, As + NH_3_ + T induces DNA damage in kidney tissue, leading to DNA strand breaks^107^. Arsenic, ammonia and high temperature enhance oxidative methylation. ROS formation leads to oxidation of DNA base lesions, DNA strand break, DNA adduct formation, and DNA protein cross link^108,109^. Indeed, Zn-NPs protect against DNA damage due to their protective role from cellular oxidation. Zn-NPs also could not involve in the oxidation–reduction reactions and protect protein sulfhydryl groups from oxidation^110,111^.

Growth performance viz. final body weight gain %, feed conversion ratio (FCR), specific growth rate (SGR), the protein efficiency ratio (PER), daily growth index (DGI %), and relative feed intake were noticeably affected by exposure to As, NH_3_ and T and concurrent with As + NH_3_ + T. Whereas supplementation of Zn-NPs improved the growth performance of the fish. The exposure to different stressors (As + NH_3_ + T) reduces the growth performance might be due to more energy demand to detoxify the ammonia and arsenic contamination in the fish. However, the energy is diverted to detoxify or remove toxins from the fish body^112^. The arsenic bioaccumulation in different parts of the fish body might not be detoxified by metabolism or any other means, which is also one of the reasons for retarding growth performance in stressors groups^22^. However, the growth performance of the fish was significantly improved with the dietary supplementation of Zn-NPs. Zn-NPs has an important role in the generation of stable associations with macromolecules, viz. nucleic acid, lipid, protein, carbohydrates and enzymes, which have a significant biological function. It also has a novel role in several enzymatic co-factor in metalloenzymes viz. RNA and DNA polymerase, carbonic anhydrase, alcohol dehydrogenase, and alkaline phosphatase. Moreover, Zn-NPs feed has higher catalytic activities, intestinal absorption and bioavailability to the fish^113^. Our earlier report demonstrated that Zn-NPs enhanced their efficiency for bioavailability and absorption in the gastrointestinal tract^21,114^. Zn also has an important role in synthesizing growth hormone in fish and enhancing somatic growth using cell division, RNA and DNA synthesis in the cell^115^. However, Zn-NPs support and improve the fish growth performance under different stressors. Gene expression of GH, GHR1, GHRβ, MYST and SMT in muscle tissue of the fish was affected by stressors (As + NH_3_ + T), whereas Zn-NPs improved the expression in fish. The growth hormone gene (GH) binds with GHR to regulate the development and growth in fish and is controlled by the hypothalamic, which regulates dopamine, ghrelin, SMT and GnRH^116,117^. Similarly, SMT and MYST gene expression in muscle tissue upregulated by stressors (As + NH_3_ + T) and downregulated with Zn-NPs. MYST depresses the myoblast, which results in terminal differentiation and division of fiber enlargement^118^. MYST and SMT, both genes, are highly regulated by mammal glucocorticoids. The information on MYST and SMT is very limited in aquatic animals such as fish.

Arsenic bioaccumulation in different tissues and concentration in water samples were studied. Arsenic was found higher in the liver tissues, followed by the kidney in the group exposed to As + NH_3_ + T. The bioaccumulation was significantly reduced with supplementation of Zn-NPs at 4 mg kg^−1^ diet. However, the results showed that Zn-NPs have the detoxifying role of As. It also enhanced the detoxification of toxin in liver and kidney tissues. The enhanced detoxification capacity of As by Zn-NPs might be due to its role in co-factor for several metabolism. Our earlier studies also reported that Zn has a strong detoxification capacity for arsenic in different fish tissues^20,21^.

## Conclusion

In the present investigation, the arsenic and ammonia toxicity and high temperature stress enhance oxidative stress, retard immunity and growth performance, alter the gene regulations and improve the bioaccumulation of arsenic in fish. Whereas dietary Zn-NPs at 4 mg kg^−1^ diet mitigated, As + NH_3_ + T stresses in *P. hypophthalmus*. In contrast to Zn-NPs at 4 mg kg^−1^ diet, the Zn-NPs at 2 and 6 mg kg^−1^ diets were not ineffective in modulating multiple stressors. In addition, in this study, we have included several stress indicators attributes and their mitigation by Zn-NPs in every aspect such as primary, secondary and tertiary stress response. Therefore, it is recommended that Zn-NPs could be used at 4 mg kg^−1^ diet to overcome the impact of multiple stresses in fish. Therefore, this data could be beneficial for formulating the feed for the culture of *P. hypophthalmus* in different agro-climatic conditions.

## Data Availability

The datasets used and/or analyzed during the current study are available from the corresponding author on reasonable request.
